# Formation of Shelf-Stable Pickering High Internal Phase Emulsion Stabilized by *Sipunculus nudus* Water-Soluble Proteins (WSPs)

**DOI:** 10.3389/fnut.2021.770218

**Published:** 2021-11-23

**Authors:** Yupo Cao, Yaping Dai, Xuli Lu, Ruyi Li, Wei Zhou, Jihua Li, Baodong Zheng

**Affiliations:** ^1^Key Laboratory of Tropical Crop Products Processing of Ministry of Agriculture and Rural Affairs, Agricultural Products Processing Research Institute, Chinese Academy of Tropical Agricultural Sciences, Zhanjiang, China; ^2^College of Food Science, Fujian Agriculture and Forestry University, Fuzhou, China; ^3^Hainan Key Laboratory of Storage and Processing of Fruits and Vegetables, Zhanjiang, China

**Keywords:** *Sipunculus nudus*, the water-soluble proteins (WSPs), the high internal phase emulsions (HIPEs), stable emulsions, environmental stability

## Abstract

To form a stable emulsion system, the water-soluble proteins (WSPs) of *Sipunculus nudus* were prepared as the sole effective stabilizer for the high internal phase emulsion (HIPEs), of which the influence of the WSPs concentration and environmental stability was investigated. The HIPEs were fabricated using a simple one-pot homogenization process (10,000 rpm/min, 3 min) that involved blending the WSPs (0.1, 1, 2, 3, 4, and 5 wt%) with soybean oil (60, 65, 70, 75, 80, 85, and 90%). The microstructure and properties of stable HIPEs were characterized by particle size, ζ-potential, visual observations, optical microscopy, and dynamic rheology property measurements. As the concentration of WSPs increases, the mean particle diameter of HIPEs decreases, on the contrary, the apparent viscosity and storage modulus gradually increase. At a given emulsifier concentration (3 wt%), the stable and gel-like HIPEs were formed at the oil internal phase (ϕ) values of 70–75%, all the pH range in values from 3 to 9, and the ionic strength from 100 to 500 mM. Furthermore, the HIPEs that were stabilized formed a gel-like state that was relatively stable to heat and storage (30 days). And there was a new phenomenon that the destabilized HIPE of the freeze-thaw treatments could still return to a gel-like state again after homogenizing. The study results suggest that the WSPs of *S. nudus* as a natural emulsifier could be widely used in the food industry.

## Introduction

High internal phase emulsions are potentially applied in food products and industries (1–7) because it has relatively high stability to droplet aggregation and creaming (8, 9). Characteristics of the high internal phase emulsion (HIPE) systems are usually a minimum internal phase volume fraction (ϕ) of 0.74 for hexagonal close packing of droplets or 0.64 for random close packing (10). Traditional stable HIPE systems were commonly stabilized by a great majority of expensive surfactants as emulsifiers (11, 12). To date, HIPEs could be stabilized by modified silica (13–15), microgels (16), titania particles (17), and hydroxyapatite (18), but among these, only a few particles are food-grade or suitable for food formulations. The particle-stabilized HIPEs have advantages in terms of environmental safety, long-term coalescence stability, and gel-like behavior compared with surfactants (16, 19, 20). The HIPEs stabilized by some natural active ingredients exposed many advantages over traditional surfactants, such as the much fewer number of stabilizers demanded, higher storage stability, and much less extent of environmental problems (21). Among them, protein-based particles appear more likely to be HIPE stabilizers because of their good emulsifying ability and ease of manufacturing (21). The first study of protein-based Pickering HIPEs was reported on pure bovine serum albumin (BSA) scaffolds from HIPEs template (22). In the previous research, Pickering HIPEs stabilized by various protein-type particles were manufactured, including prolamine (i.e., gliadin, zein) (23–27), gelatin (6, 28), whey protein (29), and native plant globular proteins (21). Pickering HIPEs, especially protein-based Pickering HIPEs have gained widespread attention in many domains, comprising food, pharmaceuticals, personal care products, as well as tissue engineering (28).

*Sipunculus nudus (S. nudus)* is a species of marine worms belonging to the phylum Sipuncula. It is widely cultivated in coastal areas globally (30, 31). *Sipunculus nudus* is rich in proteins, polysaccharides, fatty acids, and other substances. Its body is like a piece of intestine and its body length is approximately 10–20 cm, and the protein content is as high as 85% (g/g, d.b.). However, there was little research for studying the functional properties of *S. nudus* protein. In previous work, Cha et al. (32) found that the Emulsifying Activity Index (EAI), Emulsifying Stability Index (EsI), apparent viscosity, and zeta absolute potential of mussel myofibrillar protein (MMP) and lecithin stabilized emulsion increased and were in a state of good dispersion after a high-pressure homogenization treatment. These results will contribute to improving the formulation of emulsions and their properties, and provide a new understanding of MMP as a natural emulsifier in the food industry. Zou et al. (33) reported that ultrasound treatment could cause an increase in the surface hydrophobicity, fluidity, and emulsifying properties of water-soluble proteins (WSPs) in this study, which changed the advanced spatial conformation of proteins. The WSPs from chicken livers by ultrasound treatment had a higher emulsifying activity index and emulsion stability index than those without ultrasound. It is worth noting that most water-soluble proteins are used in emulsion-based foods by compounding with lecithin and other ingredients or using high-pressure homogenization and other processing techniques to improve their emulsification properties. The emulsification ability is not strong, and the complexity of the processing procedures, increased cost and other defects, and the use of protein as the only emulsifier to build a stable HIPE emulsion system by adding small doses, has not been reported. The preliminary experiment of the applicant found that the crude WSP of *S. nudus* as the sole emulsifier can form a stable oil-in-water HIPE system through a one-step homogenization method. Therefore, it is necessary to carry out the emulsion stabilized with protein to fill the study of the functional properties of *S. nudus* protein.

In this work, the main objective was to probe the potential of the WSPs from *S. nudus* as stabilizers for HIPEs. The effects of protein concentration in the continuous phase and environmental stability on the formation and properties of the HIPEs stabilized by WSPs were investigated. The test performance of the HIPEs was researched using visual observations, optical microscopy, and dynamic oscillatory measurements. This study uses WSPs as the sole stabilizer to prepare stable gel-like HIPEs, which provides a theoretical basis and technical support for studying the structural characteristics of food-grade emulsifiers and the formation mechanism of stable emulsion systems.

## Materials and Methods

### Materials

*Sipunculus nudus* samples were obtained from the Dongfeng market in Zhanjiang, Guangdong, China. Soybean oil was obtained from Wal-Mart in Zhanjiang, Guangdong, China. All other reagents arose out of analytical grade.

### Extraction and Analysis of *S. nudus* WSPs

Water-soluble proteins extraction was performed on *S. nudus* using the ammonium sulfate precipitation method according to previous research but with some modifications (25). The fresh *S. nudus* was washed, and its visceral and coelomic fluid was removed to get the body wall. The body wall of *S. nudus* was mixed with a phosphate buffer solution (PBS) (10 mM, pH 7.2) (1:2 w/v) and was minced using a meat grinder (SD-JR39, Foshan Shunde Sandi Electric Appliance Manufacturing Co., Ltd, China) at high speed gear for 30 s three times, paused for 10 s between intervals. After being minced, the meat slurry was centrifuged at 10,000 g for 15 min at 4°C (Sigma/3–30K, Sigma, Germany). After centrifugation, the supernatant was salted out and precipitated with saturated ammonium sulfate for 12 h, and dialyzed with PBS (10 mM, pH 7) for 48 h. The dialysate was lyophilized to produce protein powders. The purity of the WSPs after lyophilization was 85.3% by the method of Kjeldahl nitrogen. The mean particle sizes and ζ-potential of WSPs were 234.2 ± 25.1 nm and −22.8 ± 0.4 mV (pH: 7; salt level: 0 mM).

### Pickering HIPEs Preparation

Pickering HIPEs were fabricated using a method in the previous study (34). Briefly, aqueous proteins (0.1, 1, 2, 3, 4, and 5 wt%) in a buffer solution (0.1 mM, pH 7) containing sodium azide (0.02% w/w) as an antimicrobial agent was added to oil (75% v/v) and then homogenized at 10,000 rpm (Ultraturrax T18, IKA, Staufen, Germany) for 3 min at room temperature to form Pickering HIPEs.

### Effect of Oil Phase Fractions

In this series, the protein concentration was 3%, and the volume fractions of the oil phase were 60, 65, 70, 75, 80, 85, 90, and 95%, respectively. The Pickering emulsion for HIPEs were fabricated using the preceding emulsification procedure (section Pickering HIPEs Preparation).

### Droplet Size and Zeta Potential Measurements

The droplet size and polydispersity index (PDI) of the Pickering HIPEs were measured using a Malvern Zetasizer Nano (Malvern Instruments, United Kingdom). The refractive indexes of the oil and deionized water were 1.46 and 1.33, respectively. The diluted sample (1 mL, 0.5 mg/ml) was transferred to the quartz tube using a pipette and the droplet size was measured. The zeta potential of the Pickering HIPEs was measured by phase analysis light scattering (PALS) using Zetasizer. The electrophoretic mobility was measured and then the zeta potential was calculated with the Smoluchowski equation. All measurements were made at 25°C. The results were recorded as the average of at least three independent readings for each sample.

### Microstructure

The microstructures of the Pickering HIPEs were surveyed using an inverted microscope (Leica DMI6000 B, Leica, Germany). A drop of the emulsion sample from the bottom of the sample bottle was deposited on the microscope slide and covered with a coverslip. All the measurements were performed at room temperature.

### Rheological Properties

The rheological properties were measured using a dynamic rheometer (TA, American) with a P 35 mm steel parallel plate configuration as described by the method of Zhao et al. (35) with minor modifications. Each sample was placed between the parallel plates with a 0.5 mm gap. Dynamic strain scanning confirmed the range of the linear viscoelastic region (LVR). At a constant frequency of 1 Hz, the strain was increased logarithmically from 0.1 to 100%. Frequency sweep measurement, including the storage modulus (G′) and loss modulus (G″), was performed with the frequency increasing from 0.1 to 100 rad/s to at 1% shear strain. Shear sweeps were performed from 0.1 to 100 s^−1^ to study the change in the apparent viscosity (η) of the HIPEs with shear rate. All these rheological measurements were carried out at 25°C and performed in three replicates.

### HIPEs Stability Testing

The HIPEs samples were stored at room temperature for 0, 7, and 30 days, and the storage stability was evaluated visually to investigate creaming or coalescence. The phenomenon of cream or oil was checked and photographed by a mobile phone camera (iPhone 6, Apple, California, United States) during storage.

### Environmental Stability of HIPEs

The stability of the HIPEs stabilized by WSPs was subjected to environmental stresses that emulsion-based food products might encounter in commercial applications, such as pH variations, salt addition, heating, or freeze-thawing treatment at ambient temperature. In all the emulsions prepared, the protein concentration and the volume fractions of oil were 3 and 75%, respectively.

pH-stability: The effect of pH (3–9) on the properties of the HIPEs was investigated. The pH value was adjusted by adding different amounts of hydrochloric acid (HCl) or sodium hydroxide (NaOH) solution.

Salt-stability: The emulsion was adjusted to different salt levels [0–500 mM sodium chloride (NaCl)] by adding NaCl, and then transferred into a glass sample bottle.

Thermal stability: The effect of the HIPE properties under different heat treatment (40–100°C) conditions were investigated. Briefly, the HIPEs were held for 30 min at different temperatures and then cooled down to 25°C.

Freeze-thawing stability: To verify the freeze-thaw stability, the HIPEs were frozen at −20°C for 24 h and then thawed at 25°C for 4 h. The appearance and microstructure of the fresh or treated HIPEs were characterized by visual observation and optical microscopy, as described above.

### Statistical Analysis

All analyses were performed in triplicate. The experimental results were expressed as means ± SD. Statistical analysis was conducted using Origin 8.5 and SPSS 16.0 (IBM, Armonk, New York, United States). The data were analyzed by ANOVA (*p* < 0.05) and the means was separated by Duncan's multiple range test.

## Results and Discussion

### Formation and Characteristics of HIPEs Stabilized by WSPs

**Figure 1:**
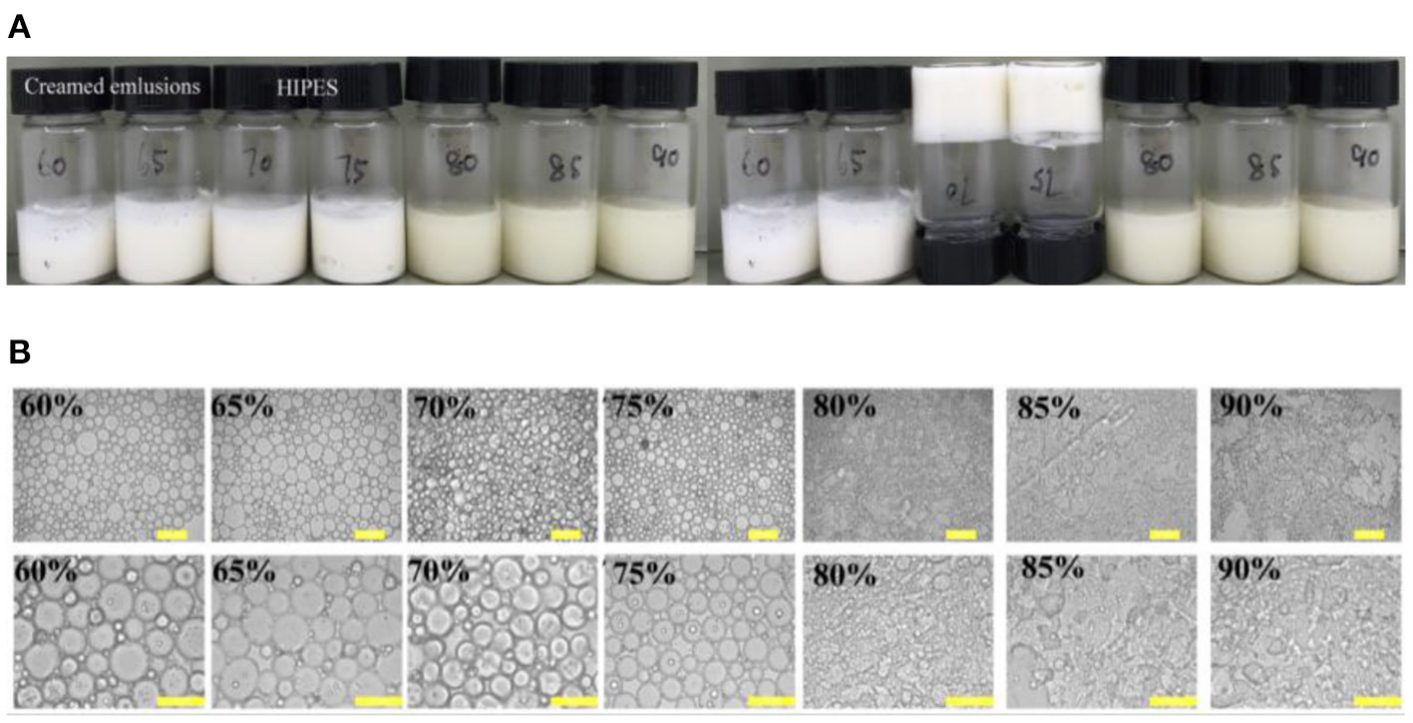
Visual appearance **(A)** and optical micrographs **(B)** of freshly prepared water-soluble protein (WSP)-stabilized emulsion by the protein at a constant solid concentration (3 wt%) in the aqueous phase, but varying soy oil fractions ϕ values of 60–90%. The pH and salt concentrations were 7 and 0 mM, respectively. The scale bars are 100 and 50 μm, respectively.

The visual appearance and optical micrographs of the freshly prepared WSP-stabilized emulsion by the protein at a constant protein concentration (3 wt%) and oil phase ϕ values of 60–90% were recorded in Figure 1. Traditionally, extensive surfactants are often demanded to availably perform table HIPEs (36). As expected, the emulsion suffered from creaming slowly for the dispersed phase fractions lower than 70%. This is unanimous with the fact that when soy oil fractions ϕ values increased above 70%, droplets begin to deform due to the close packing or close hexagonal packing in the emulsion (37, 38). However, for internal phases between 70 and 75%, the emulsion exhibited a gel-like behavior and marked stability against coalescence, and if the ϕ values were higher than 75%, catastrophic phase separation occurred (Figure 1A). Similar results have been reported for the HIPEs stabilized by protein/polysaccharide hybrid particles (39) and assembled block copolymer (3). The optical microscopic images of these emulsions showed that the droplet size gradually decreased when the soy oil fractions ϕ values increased from 60 to 75%, and it cannot form the emulsion droplet at ϕ>75% (Figure 1B). This result reflected that WSPs are suitable for stabilizing Pickering HIPEs, but a stable emulsion system cannot be generated when a fewer amount of proteins are incapable to cover the interfacial area during the emulsification. With respect to that, HIPEs are usually obtained by a suitable internal ϕ value of 74% (14, 40). Therefore, most of the emulsion droplets were generally engendered with internal volume fraction of up to 70 or 75%, which has been similarly discovered for the HIPEs stabilized by meat protein particles (41) and protein-pectin compounds (5).

**Figure 2:**
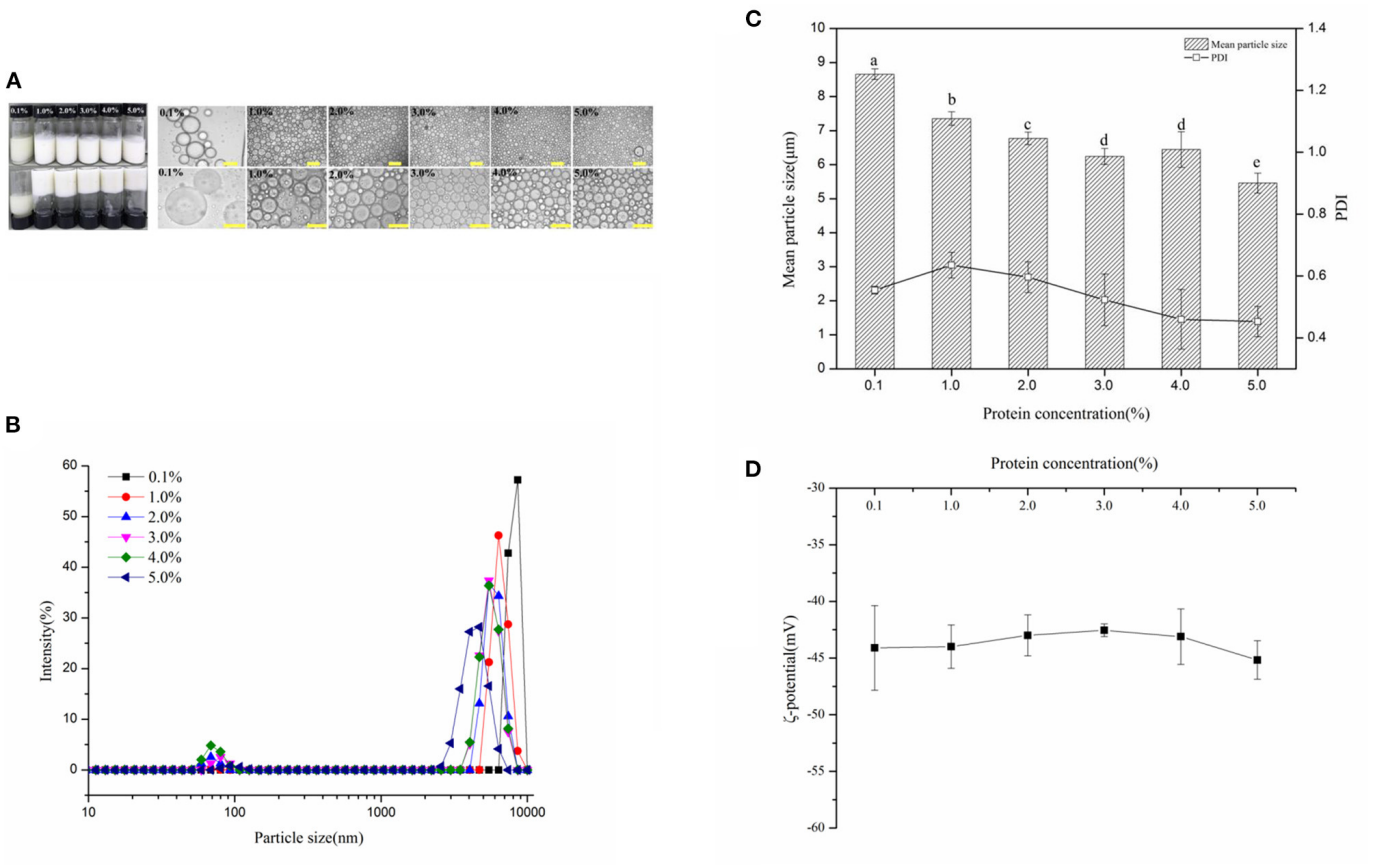
**(A)** Visual appearance and optical micrographs of fresh WSP-stabilized high internal phase emulsions (HIPEs) (oil fraction, ϕ = 75%), at varying protein concentration c values of 0.1, 1, 2, 3, 4, and 5%, respectively. The scale bars are 100 and 50 μm, respectively. **(B)** The distribution of the HIPE droplet size in the aqueous phase with varying protein concentrations. The mean particle sizes and polydispersity indexes (PDI) **(C)** and ζ-potential **(D)** of HIPEs prepared by WSP (0.1, 1, 2, 3, 4, and 5 wt%). Different letters (a, b, c, d, e) in the same column indicate significant differences (*p* < 0.05.)

Figure 2A showed the visual appearance and optical micrographs of fresh WSP-stabilized HIPEs (oil fraction, ϕ = 75%), at varying protein concentration c values of 0.1–5 wt%. It is noteworthy that the gel-like emulsion could not form as the c values of 0.1% and showed creaming and phase separation. The appearance change of the HIPEs was not observed as the WSP concentration enhancement from 1 to 5 wt% and exhibited gel-like behavior. The optical microscopic observations exhibited that the droplet size of these HIPEs reduced with enhancing the c in the range 1–5 wt%. In addition, the change of the mean particle sizes with WSP concentrations was also characterized in Figures 2B,C. It can be observed that their mean particle sizes decreased from around 8.66–5.46 μm while the protein concentration increased from 0.1 to 5 %, wherein the PDI ranged from 0.4 to 0.6. It suggested that the particle concentration in the continuous phase plays a crucial role in the hydrogel network formation in these stabilized HIPEs (42). Interestingly, the absolute value of the ζ-potential (Figure 2D) of the HIPEs was stable at 45 ± 5 mV and the potential value slightly increased with the increase of protein concentration. This result suggested that the increase of protein concentration contributed to the electrophoretic signal used to calculate the ζ-potential (43). The relatively high negative charge probably accounts for their good physical stability since it will lead to a strong electrostatic repulsion between them (44).

**Figure 3:**
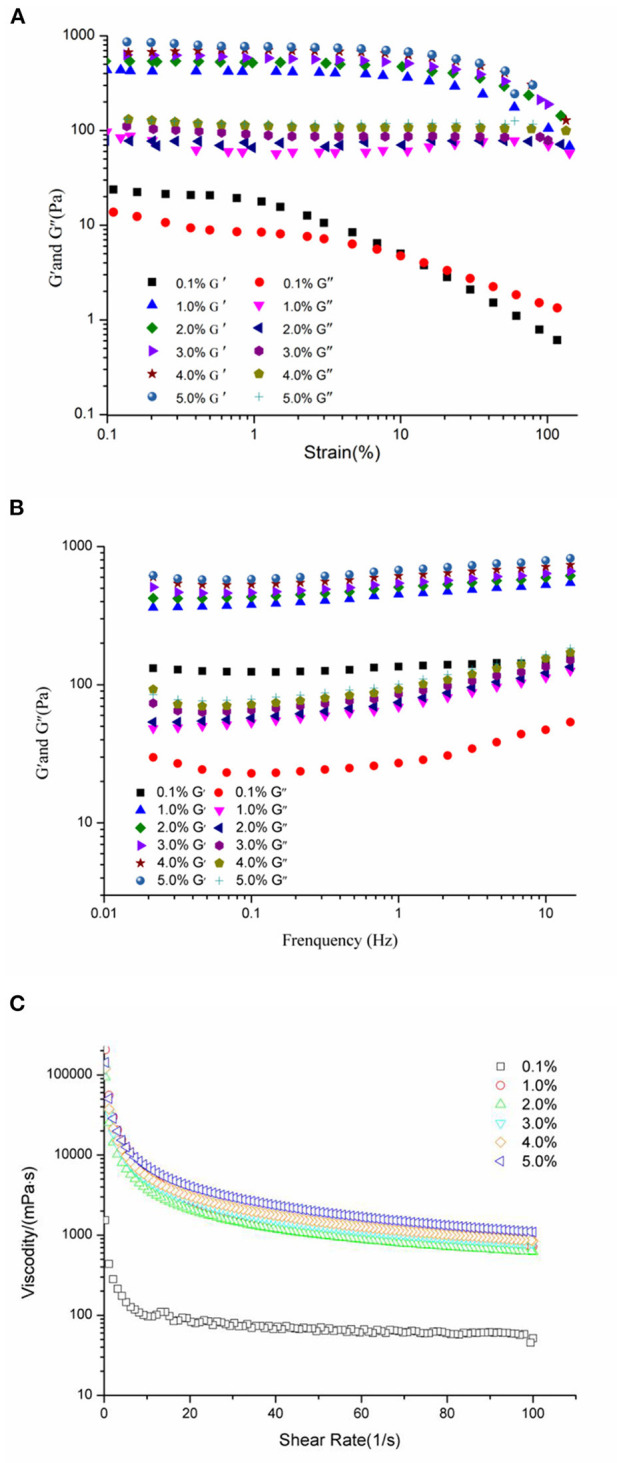
Rheological properties of HIPEs prepared by WSP (0.1, 1, 2, 3, 4, and 5 wt%). Strain sweep **(A)** of the HIPEs with strain increased from 0.1 to 100% at a constant frequency of 1 Hz. Frequency sweeps **(B)** curves at fixed strain (1%) with the frequency ranging from 0.01 to 15 Hz; Apparent viscosity **(C)** of the HIPE samples with shear rate from 0 to 100 1/s.

Rheological measurements provided valuable information about the properties and performance of food emulsions (45, 46). The relationship between WSP concentrations and the gel-like structure of HIPEs is shown in Figure 3. The strain sweep (Figure 3A) of the HIPEs indicated that the G′ and G″ were independent of the strain at the low strain amplitude, and the G″ was lesser than the G′, which demonstrated that all of the HIPEs behaved elastic and gel-like (25, 47). Furthermore, the G′ and G″ gradually decreased with the increase of the WSP concentration at the same strain. These results show that the smaller droplets of emulsions had higher G′, which is also accordant with the existing finding (48). It is observed that with the increase of strain amplitude, G′ and G″ did not change significantly, and then decreases slightly, so as to further determine the strain force. The frequency sweep tests (Figure 3B) were executed at a fixed strain (1%) with the frequency ranging from 0.01 to 15 Hz. As can be seen from the frequency sweeps curves, the G′ was always higher than G″ for the WSP-stabilized HIPEs, which represents viscoelastic solid behavior and a high gel-like strong structure. Both G′ and G″ presented slight increases with the raise of protein concentrations in the emulsion, in accordance with the HIPEs stabilized by WSP (0.1–5% w/w). There were some similar findings with soy β-conglycinin (10) and whey protein (49). As shown in Figure 3C, the apparent viscosity depressed evidently with the enhancing the shear rate, and the increase of the protein particle concentration resulted in higher viscosity, especially, the viscosity decreased significantly compared with other concentrations when the protein concentration was 0.1%. This influence suggested that the effect of the WSP concentration on the rheology of the HIPEs was chiefly owed to the larger contact surface area between the oil droplets at higher WSP concentrations, rather than the free flow of HIPEs in a shear field (50). According to Stoke's law, the improved viscosity of emulsion would help to slow down the droplet floating speed and maintain better stability (51).

### Effects of pH on Properties and Stability of the HIPEs

**Figure 4:**
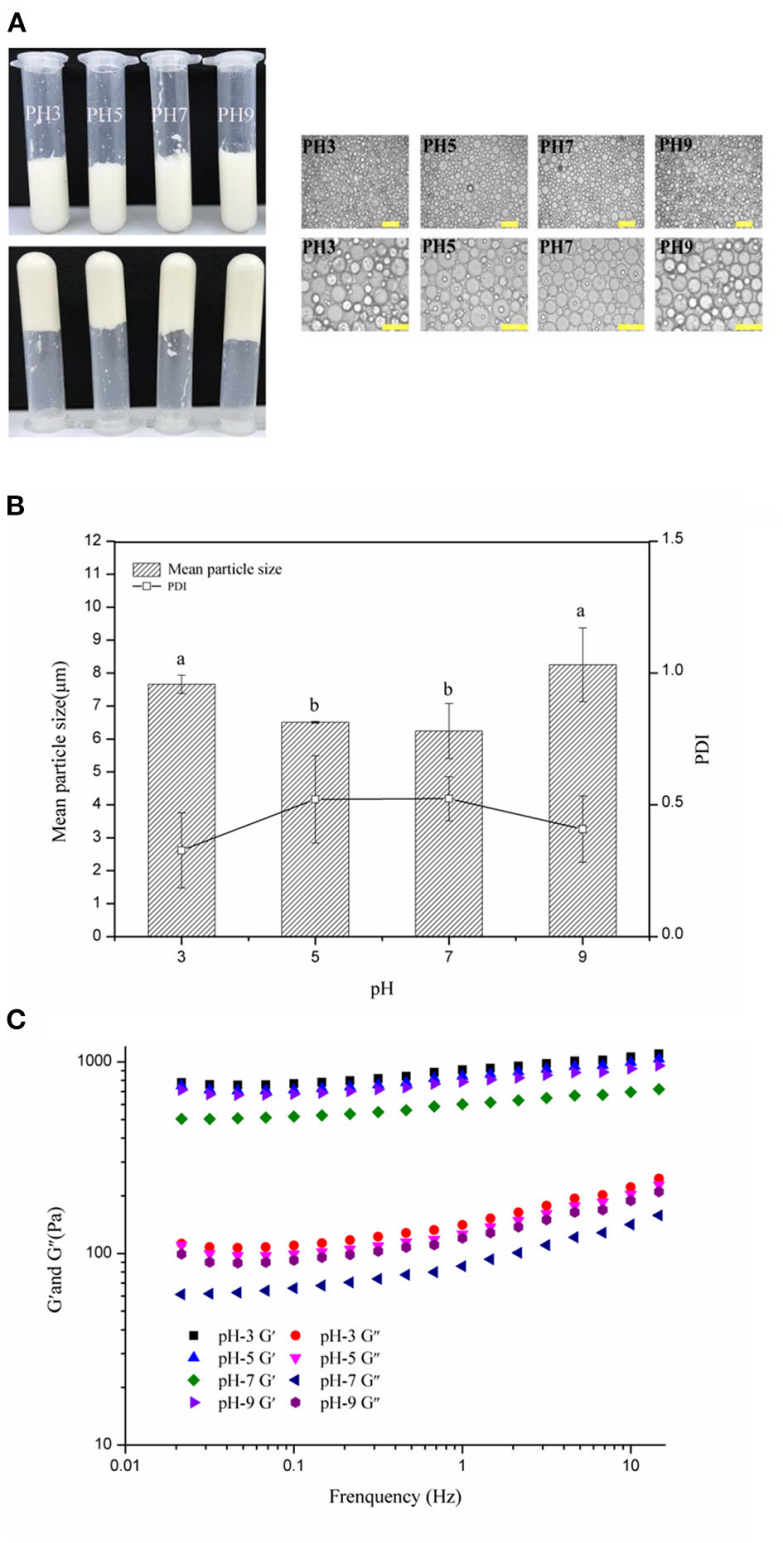
Visual appearance and optical micrographs **(A)**, mean particle sizes and PDI **(B)**, frequency sweeps **(C)** of the WSP-stabilized HIPEs at pH values of 3–9. Different letters (a, b) in the same column indicate significant differences (*p* < 0.05).

According to previous reports, some food-grade particles required specific pH conditions to form stable HIPEs such as zein-propylene glycol alginaterhamnolipid complex particles (pH 5) (23), gelatine particles (pH 12) (6), and soy glycinin (pH 7) (52), which might limit the application of HIPEs. Therefore, in this study, the visual appearance, optical micrographs, frequency sweeps, mean particle sizes, and PDI of the HIPEs (c: 3 wt%; ϕ: 75%) with different pH values (3–9) were measured to analyze the limited pH values of the WSP-stabilized HIPEs. With the visual appearance (Figure 4A, left), stable HIPEs were formed from disparate pH values of the water phase. Moreover, the samples did not flow when they were inverted, denoting that they preserved their mechanical integrity. The optical micrographs images (Figure 4A, right) and mean particle sizes (Figure 4B) showed that the distribution of the droplet sizes of the HIPEs ranged from 1 to 10 μm (PDI: 0.40–0.5), and the droplet size of the HIPEs slightly reduced under the acidic or alkaline conditions. It can be observed that the droplet size of the WSP-stabilized HIPEs was the smallest at pH 7 and five of the WSP-stabilized HIPEs had almost the same droplet size. When pH is higher than 7, the droplet size increased with the increase of the pH value. In contrast, if the pH value is lower than 5, the droplet size increased with the decrease of the pH value. This may be because of the impairment of the emulsification ability, which could be due to the solubility of WSPs in stronger acidic or alkaline conditions or the change of electrostatic repulsion, resulting in the flocculation of emulsion droplets. This was further supported by the results of the rheology measurements. In Figure 4C, the HIPEs had variant dynamic rheology properties under different pH conditions. When the pH was 3, the elastic modulus values of the HIPEs were higher than that of other treatments. These phenomena manifested that the rheological properties of the emulsion had a certain degree of change with the pH value of the aqueous phase (53, 54). This suggested that the intensity of protein interactions at different pH values is sensitively incarnated in the rheological properties of the emulsion (55, 56). However, the characteristics of protein on the emulsion interfacial film are not yet clear, and further studies are needed (57).

### Effects of Salt Levels on Properties and Stability of the HIPEs

**Figure 5:**
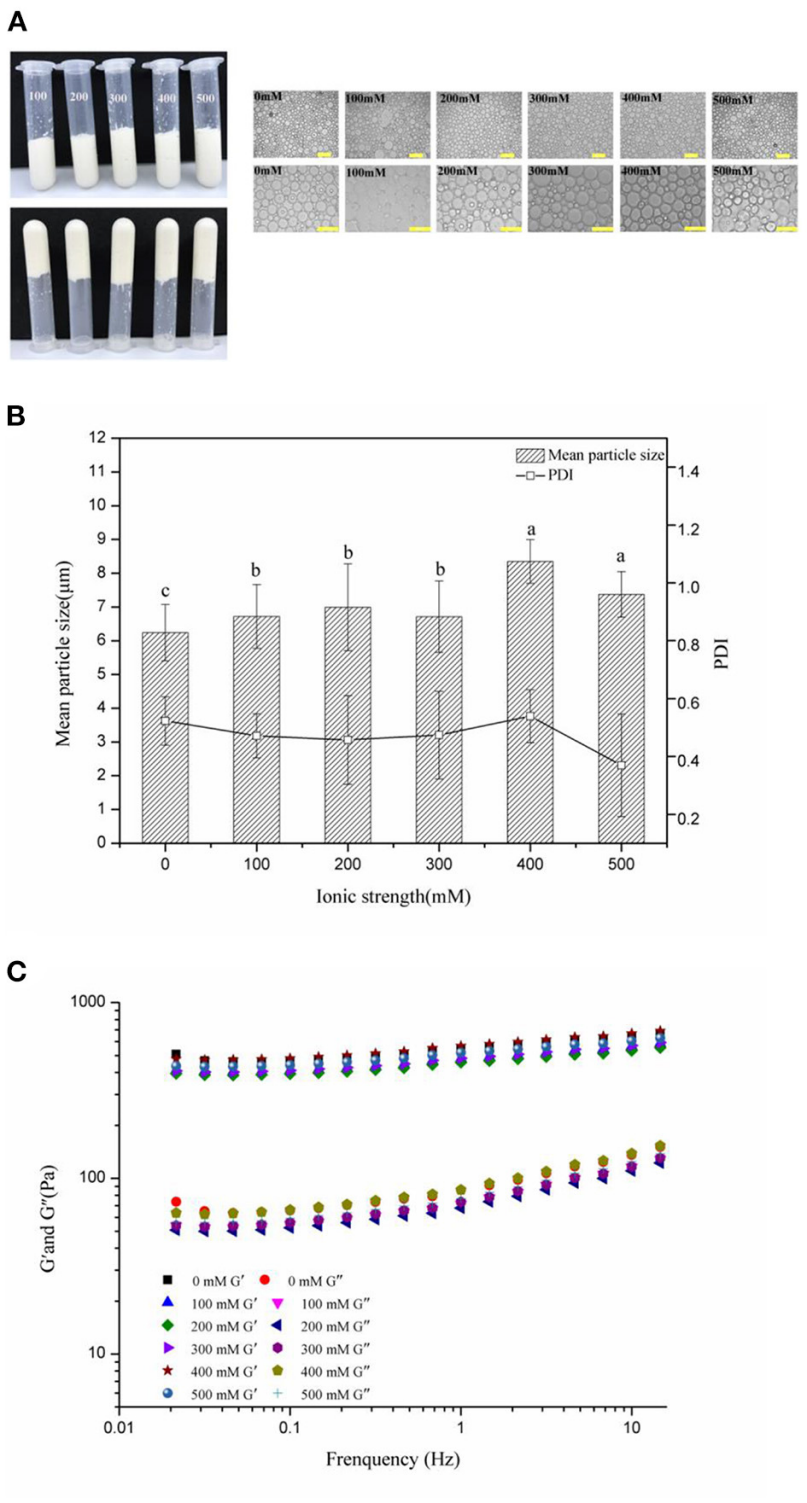
Visual appearance and optical micrographs **(A)**, mean particle sizes and PDI **(B)**, frequency sweeps **(C)** of the WSP-stabilized HIPEs at different salt concentrations. Different letters (a, b, c) in the same column indicate significant differences (*p* < 0.05).

Figure 5 exhibits the visual appearance and optical micrographs, mean particle sizes, and PDI of the frequency sweeps of the WSP-stabilized HIPEs by 3 wt% of WSPs, at pH 7 and varying salt levels of 0–500 mM. In the visual appearance picture (Figure 5A), the HIPEs had good stability at all salt levels, there was no alteration in their visual appearance and they did not flow when they were inverted. These results indicated that the Pickering HIPEs stabilized by WSPs could be formed over a range of salt concentrations, which may be because steric repulsion was the dominant force opposing droplet aggregation, rather than electrostatic repulsion (58). From the measurement of the droplet size (Figure 5B), the mean particle sizes of all these HIPEs ranged from 5 to 10 μm, and the droplet size enlarged with the enhancement of the NaCl concentration. It suggests that increasing the ionic levels would raise the size of the emulsion droplet (41). Finally, frequency sweeps (Figure 5C) showed that the G′ was always higher than G″ for all of the WSP-stabilized HIPEs, further explaining the viscoelastic solid behavior and high gel-like strength structure at all salt concentration levels. Li et al. (34) detected similar phenomena for HIPEs stabilized by marine Antarctic krill protein. And the elastic moduli of HIPEs did not depend strongly on salt levels. These results were consistent with the visual observations, suggesting that electrostatic interactions did not play a major role in holding the gel structure of the HIPEs together (59).

### Stability of HIPEs Storage, Heating, and Freeze-Thawing

**Figure 6:**
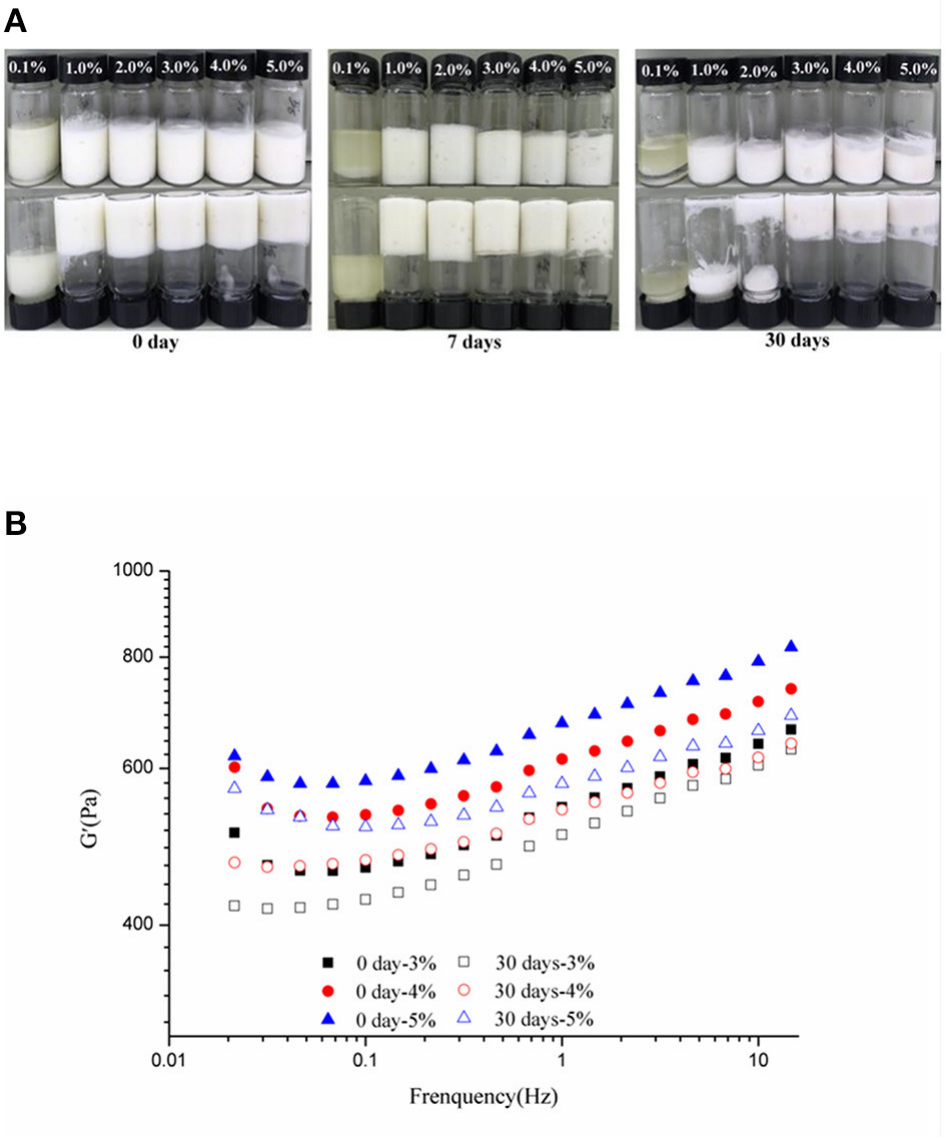
Visual appearance (c: 0.1, 1, 2, 3, 4, and 5%) **(A)** and frequency sweeps (c: 3–5%) **(B)** of the WSP-stabilized HIPEs during storage at 0, 7, and 30 days, respectively.

The stability of the HIPEs by WSPs to the c values of 0.1–5 wt% elongated storage (up to 0, 7, and 30 days) was evaluated, using visual observations and rheological measurements. As shown in Figure 6A, the visual appearance of the HIPEs did not suffer a noticeable change after storage up to 30 days at the c values of 3–5 wt%. At the c values of 1 and 2 wt%, the formed Pickering HIPEs were in a liquid state after storage up to 30 days, though the HIPEs were kinetically stable in storage up to 7 days. However, when the c was 0.1 wt%, unstable emulsion with severe oiling off was observed, which was consistent with the results observed in Figure 2A. In addition, Figure 6B described that the frequency sweeps of the WSP-stabilized HIPEs of the c values of 3, 4, and 5 wt% during storage at 0 and 30 days, respectively, and their G′ distinctly decreased after storage. Xu et al. (21) detected similar phenomena for HIPEs stabilized by soy β-conglycinin.

**Figure 7:**
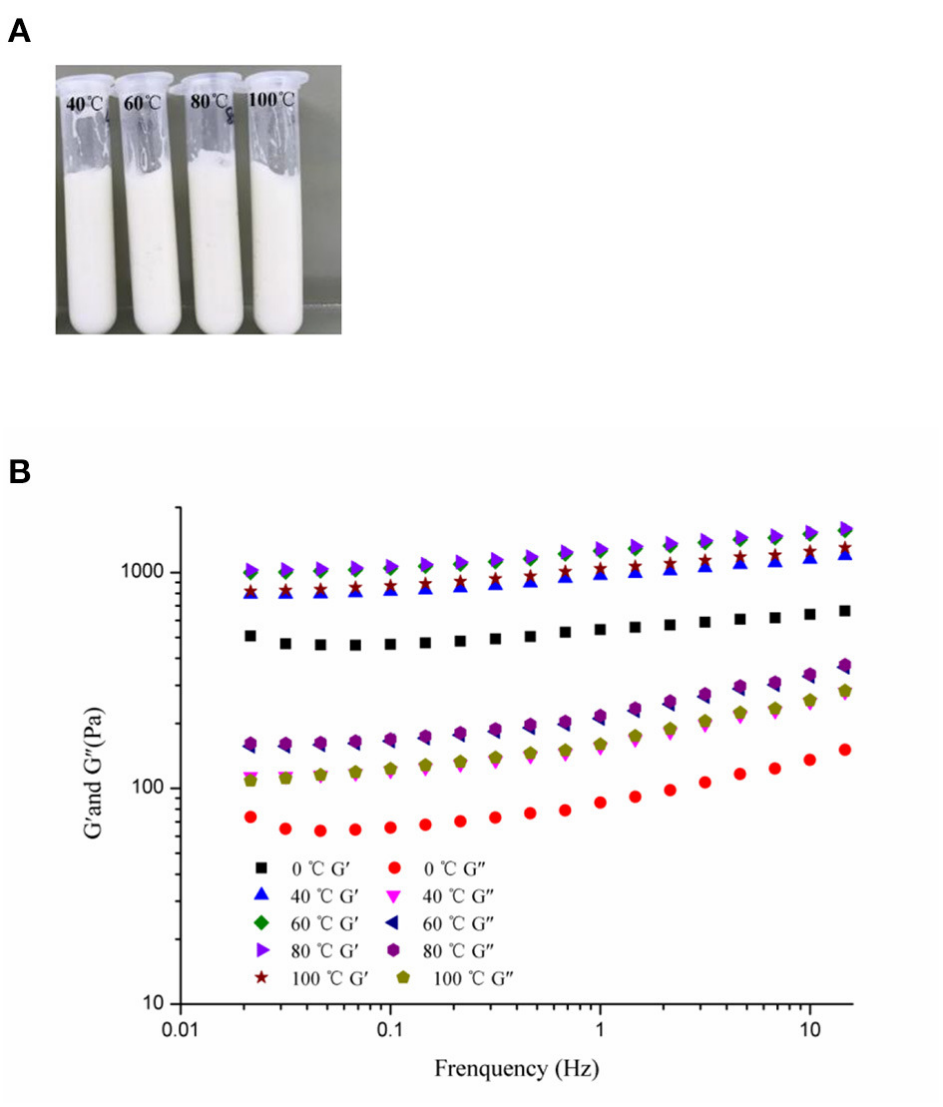
Visual appearance **(A)** and frequency sweeps **(B)** of the WSP-stabilized HIPEs as unheated, heated at 40, 60, 80, and 100°C, respectively.

**Figure 8:**
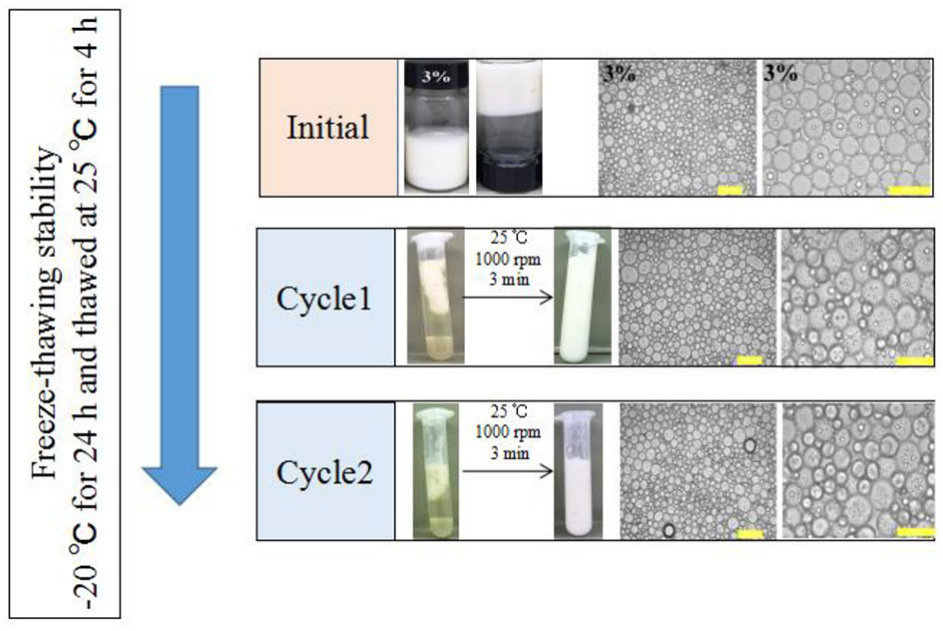
Visual appearance and optical micrographs of the WSP-stabilized HIPEs at a fixed c value of 3 wt%, before or after two cycles of freeze-thaw treatment. The scale bars are 50 and 100 μm, respectively. All the HIPEs were frozen at −20°C for 24 h and then thawed at 25°C for 2 h. In all cases, the same emulsification process was performed using a one-pot homogenization at 10,000 rpm for 3 min at room temperature.

Foods are usually subjected to thermal processes during their manufacturing or utilization (60). On this account, the influence of heat treatment (at 40, 60, 80, and 100°C for 30 min and then cooled to 25°C) on the stability of the samples was implemented. Figure 7A showed that the visual appearance of all test HIPEs did not change under heating. Moreover, according to Figure 7B, the HIPEs had more stronger elasticity after heating, which could be because the protein was denatured to form a tighter network structure after heating treatment (61). In the food industry, oil-in-water emulsion with good freeze-thaw stability has many potential applications (41). In this research, the HIPEs were tested with a freeze-thawing treatment (freeze: −20°C for 24 h; thaw: room temperature for 2 h) (Figure 8). It should be noted that after one cycle of freeze-thawing treatment, all the HIPEs appeared to have a phase separation occurring from the visual appearance. Surprisingly, in the optical microscope image, the phenomenon was found that the destabilized HIPE of the freeze-thaw treatments could still return to a gel-like state again after homogenizing at 10,000 rpm for 3 min at room temperature. The cycle of the freeze-thaw treatment indicated that these HIPEs exhibited extraordinary temperature responsiveness.

## Conclusions

In this work, the WSPs from the body wall of *S. nudus* were firstly studied if they will successfully form and stabilize Pickering HIPEs. The results showed that the stable Pickering HIPEs could be easily formed at ϕ values in the range 70–75% by the protein at a constant solid concentration (3 wt%). In addition, the all stable and gel-like Pickering HIPEs could be engendered at the c value of the WSP concentration in the range 1.0–5.0 wt%, and the apparent viscosity and shear modulus of the HIPEs increased with the increase of the protein concentration. Under strong acid or base conditions, the particle size and dynamic rheology property of HIPEs will be affected to some extent, but the gel-like emulsion could be formed in all of the pH range (3–9) in the continuous phase. Similarly, the HIPEs had good stability at all salt levels, the mean particle sizes of all these HIPEs ranged from 5 to 10 μm which enlarged with the enhancement of the ionic strength. All formed HIPEs represented excellent stability upon heating or freeze-thawing after homogenizing again. The visual appearance of the HIPEs did not suffer a noticeable change after storage up to 30 days at the c values of 3–5 wt%. This study suggests that the WSPs of the *S. nudus* could exhibit great potential for applications in forming stable HIPEs. It will provide a basis for further study of the specific interfacial architecture and the stability mechanism of the emulsion of HIPEs stabilized by the WSPs particles.

## Data Availability Statement

The original contributions presented in the study are included in the article/supplementary material, further inquiries can be directed to the corresponding authors.

## Author Contributions

JL participated in the conception of the idea and provided the resources for the development of the study. BZ participated in the study design and supervised the analyses. XL and RL participated in the performance of analyses. YC and YD participated in data analysis and collection, as well as writing the original manuscript. WZ performed the statistical analysis. All authors contributed to the article and approved the submitted version.

## Funding

This research was funded by the Natural Science Foundation of Hainan Province of China (Grant Number: 320MS092; 321QN305 ) and Central Public-interest Scientific Institution Basal Research Fund for the Chinese Academy of Tropical Agricultural Sciences (Grant Number: 1630122017023).

## Conflict of Interest

The authors declare that the research was conducted in the absence of any commercial or financial relationships that could be construed as a potential conflict of interest.

## Publisher's Note

All claims expressed in this article are solely those of the authors and do not necessarily represent those of their affiliated organizations, or those of the publisher, the editors and the reviewers. Any product that may be evaluated in this article, or claim that may be made by its manufacturer, is not guaranteed or endorsed by the publisher.

## References

[1] Kim K Kim S Ryu J Jeon J Jang SG Kim H. Processable high internal phase pickering emulsions using depletion attraction. Nat Commun. (2017) 8:14305. 10.1038/ncomms14305 28145435 PMC5296636

[2] Butler R Davies CM Cooper AI. Emulsion templating using high internal phase supercritical fluid emulsions. Adv Mater. (2001) 13:1459–63. 10.1002/1521-4095(200110)13:19<1459::AID-ADMA1459>3.0.CO;2-K

[3] Zhang T Xu Z Wu Y Guo Q. Assembled block copolymer stabilized high internal phase emulsion hydrogels for enhancing oil safety. Ind Eng Chem Res. (2016) 55:4499–505. 10.1021/acs.iecr.6b00039

[4] Chan-Ik P Wan-Gu C Seong-Jae L. Emulsion stability of cosmetic creams based on water-in-oil high internal phase emulsions. Korea Aust Rheol J. (2003) 15:125–30. 10.1016/S0167-6105(03)00080-1

[5] Wijaya W Van-der Meeren P Dewettinck K Patel AR. High internal phase emulsion (HIPE)-templated biopolymeric oleofilms containing an ultra-high concentration of edible liquid oil. Food Funct. (2018) 9:1993–7. 10.1039/C7FO01945A 29560481

[6] Tan H Tu Z Jia H Gou X Ngai T. Hierarchical porous protein scaffold templated from high internal phase emulsion costabilized by gelatin and gelatin nanoparticles. Langmuir. (2018) 34:4820–9. 10.1021/acs.langmuir.7b04047 29631405

[7] Zang D Clegg PS. Relationship between high internal-phase Pickering emulsions and catastrophic inversion. Soft Matter. (2013) 9:7042–8. 10.1039/c3sm00133d

[8] Liu W Gao HX McClements DJ Zhou L Wu J Zou LQ. Stability, rheology, and β-carotene bioaccessibility of high internal phase emulsion gels. Food Hydrocoll. (2019) 88 210–7. 10.1016/j.foodhyd.2018.10.012

[9] Liu X Huang YQ Chen XW Deng ZY Yang XQ. Whole cereal protein-based Pickering emulsions prepared by zein-gliadin complex particles. J Cereal Sci. (2019) 87:46–51. 10.1016/j.jcs.2019.02.004

[10] Xu YT Tang CH Binks BP. High internal phase emulsions stabilized solely by a globular protein glycated to form soft particles. Food Hydrocoll. (2020) 98:105254. 10.1016/j.foodhyd.2019.105254

[11] Barbetta A Cameron NR. Morphology and surface area of emulsion derived (PolyHIPE) solid foams prepared with oil-phase soluble porogenic solvents: span 80 as surfactant. Macromolecules. (2004) 37:3188–201. 10.1021/ma0359436

[12] Huang XN Zhou FZ Yang T Yin SW Tang CH Yang XQ. Fabrication and characterization of Pickering High Internal Phase Emulsions (HIPEs) stabilized by chitosan-caseinophosphopeptides nanocomplexes as oral delivery vehicles. Food Hydrocoll. (2019) 93:34–45. 10.1016/j.foodhyd.2019.02.005

[13] Arditty S Schmitt V Giermanska-Kahn J Leal-Calderon F. Materials based on solid-stabilized emulsions. J Colloid Interface Sci. (2004) 275:659–64. 10.1016/j.jcis.2004.03.001 15178300

[14] Ikem VO Menner A Bismarck A. High internal phase emulsions stabilized solely by functionalized silica particles. Angew Chem Int Ed. (2008) 47:8277–9. 10.1002/anie.200802244 18814159

[15] Destribats M Faure B Birot M Babot O Schmitt V Backov R. Tailored silica macrocellular foams: combining limited coalescence-based Pickering emulsion and sol–gel process. Adv Funct Mater. (2012) 22:2642–54. 10.1002/adfm.20110256425855820

[16] Li Z Ming T Wang J Ngai T. High internal phase emulsions stabilized solely by microgel particles. Angew Chem Int Ed. (2009) 48:8490–3. 10.1002/anie.200902103 19798705

[17] Ikem VO Menner A Bismarck A. High-porosity macroporous polymers sythesized from titania-particle-stabilized medium and high internal phase emulsions. Langmuir. (2010) 26:8836–41. 10.1021/la9046066 20151659

[18] Wang AJ Paterson T Owen R Sherborne C Dugan J Li JM. Photocurable high internal phase emulsions (HIPEs) containing hydroxyapatite for additive manufacture of tissue engineering scaffolds with multi-scale porosity. Mater Sci Eng C. (2016) 67:51–8. 10.1016/j.msec.2016.04.087 27287098

[19] Capron I Cathala B. Surfactant-free high internal phase emulsions stabilized by cellulose nanocrystals. Biomacromolecules. (2013) 14:291–6. 10.1021/bm301871k 23289355

[20] Chen QH Zheng J Xu YT Yin SW Liu F Tang CH. Surface modification improves fabrication of Pickering high internal phase emulsions stabilized by cellulose nanocrystals. Food Hydrocoll. (2018) 75:125–30. 10.1016/j.foodhyd.2017.09.005

[21] Xu YT Liu TX Tang CH. Novel pickering high internal phase emulsion gels stabilized solely by soy β-conglycinin. Food Hydrocoll. (2019) 88:21–30. 10.1016/j.foodhyd.2018.09.031

[22] Li ZF Xiao M Wang JF Ngai T. Pure protein scaffolds from Pickering high internal phase emulsion template. Macromol Rapid Commun. (2013) 34:169–74. 10.1002/marc.201200553 23060090

[23] Dai L Yang S Wei Y Sun C McClements DJ Mao L. Development of stable high internal phase emulsions by pickering stabilization: utilization of zein-propylene glycol alginate-rhamnolipid complex particles as colloidal emulsifiers. Food chem. (2019) 275:246–54. 10.1016/j.foodchem.2018.09.122 30724194

[24] Zhou FZ Huang XN Wu ZL Yin SW Zhu JH Tang CH. Fabrication of zein/pectin hybrid particles stabilized Pickering high internal phase emulsions (HIPEs) with robust and ordered interface architecture. J Agric Food Chem. (2018) 66:11113–23. 10.1021/acs.jafc.8b03714 30272970

[25] Zou Y Yang X Scholten E. Tuning particle properties to control rheological behavior of high internal phase emulsion gels stabilized by zein/tannic acid complex particles. Food Hydrocoll. (2019) 89:163–70. 10.1016/j.foodhyd.2018.10.037

[26] Zhou FZ Zeng T Yin SW Tang CH Yuan DB Yang XQ. Development of antioxidant gliadin particle stabilized Pickering high internal phase emulsions (HIPEs) as oral delivery systems and the *in vitro* digestion fate. Food Funct. (2018) 9:959–70. 10.1039/C7FO01400G 29322140

[27] Zhou FZ Yu XH Zeng T Yin SW Tang CH Yang XQ. Fabrication and characterization of novel water-insoluble protein porous materials derived from Pickering high internal-phase emulsions stabilized by gliadin-chitosan-complex particles. J Agric Food Chem. (2019) 67:3423–31. 10.1021/acs.jafc.9b00221 30835109

[28] Huang XN Zhu JJ Xi YK Yin SW Ngai T Yang XQ. Protein-based pickering high internal phase emulsions as nutraceutical vehicles of and the template for advanced materials: a perspective paper. J Agric Food Chem. (2019) 67:9719–26. 10.1021/acs.jafc.9b03356 31398015

[29] Su JL Wang XQ Li W Chen LG Zeng XX Huang QR. Enhancing the viability of *Lactobacillus plantarum* as probiotics through Encapsulation with the high internal phase emulsions stabilized with whey protein isolate microgels. J Agric Food Chem. (2018) 66:12335–43. 10.1021/acs.jafc.8b03807 30380846

[30] Zhang CX Dai ZR. Anti-hypoxia activity of a polysaccharide extracted from the *Sipunculus nudus* L. Int J Biol Macromol. (2011) 49:5236. 10.1016/j.ijbiomac.2011.06.018 21723315

[31] Su J Jiang L Wu J Liu Z Wu Y. Anti-tumor and anti-virus activity of polysaccharides extracted from *Sipunculus nudus*(SNP) on Hepg2.2.15. Int J Biol Macromol. (2016) 87:597–602. 10.1016/j.ijbiomac.2016.03.022 26987430

[32] Cha Y Shi X Wu F Zou H Chang C Guo Y. Improving the stability of oil-in-water emulsions by using mussel myofibrillar proteins and lecithin as emulsifiers and high-pressure homogenization. J Food Eng. (2019) 258:1–8. 10.1016/j.jfoodeng.2019.04.009

[33] Zou Y Shi H Chen X Xu P Jiang DI Weimin X. Modifying the structure, emulsifying and rheological properties of water-soluble protein from chicken liver by low-frequency ultrasound treatment. Int J Biol Macromol. (2019) 139:810–7. 10.1016/j.ijbiomac.2019.08.062 31401277

[34] Li Y Zeng QH Liu G Chen X Zhu Y Liu H. Food-grade emulsions stabilized by marine Antarctic krill (*Euphausia superba*) proteins with long-term physico-chemical stability. LWT Food Sci Technol. (2020) 128:109492. 10.1016/j.lwt.2020.109492

[35] Zhao X Wu T Xing T Xu XL Zhou G. Rheological and physical properties of O/W protein emulsions stabilized by isoelectric solubilization/precipitation isolated protein: the underlying effects of varying protein concentrations. Food Hydrocoll. (2019) 95: 580–9. 10.1016/j.foodhyd.2018.03.040

[36] Akartuna I Studart AR Tervoort E Gauckler LJ. Macroporous ceramics from particle-stabilized emulsions. Adv Mater. (2008) 20:4714–8. 10.1002/adma.20080188825855820

[37] Cohen-Addad S H?hler R. Rheology of foams and highly concentrated emulsions. Curr Opin Colloid Interface Sci. (2014) 19:536–48. 10.1016/j.cocis.2014.11.003

[38] Silverstein MS. PolyHIPEs: recent advances in emulsion-templeted porous polymers. Prog Polym Sci. (2014) 39:199–234. 10.1016/j.progpolymsci.2013.07.003

[39] Zeng T Wu ZL Zhu JY Yin SW Tang CH Wu LY. Development of antioxidant Pickering high internal phase emulsions (HIPEs) stabilized by protein/polysaccharide hybrid particles as potential alternative for PHOs. Food Chem. (2017) 231:122–30. 10.1016/j.foodchem.2017.03.116 28449988

[40] Yang T Zheng J Zheng BS Liu F Wang S Tang CH. High internal phase emulsions stabilized by starch nanocrystals. Food Hydrocoll. (2018) 82:230–8. 10.1016/j.foodhyd.2018.04.00634587529

[41] Li R He Q Guo M Yuan J Wu Y Wang S. Universal and simple method for facile fabrication of sustainable high internal phase emulsions solely using meat protein particles with various pH values. Food Hydrocoll. (2019) 100:105444. 10.1016/j.foodhyd.2019.105444

[42] Hunter TN Pugh RJ Franks GV Jameson GJ. The role of particles in stabilising foams and emulsions. Adv Colloid Interface Sci. (2008) 137:57–81. 10.1016/j.cis.2007.07.007 17904510

[43] Pei Y Wan J You M McClements DJ Li Y Li B. Impact of whey protein complexation with phytic acid on its emulsification and stabilization properties. Food Hydrocoll. (2019) 87:90–6. 10.1016/j.foodhyd.2018.07.034

[44] Li R Dai T Tan Y Fu G Wan Y Liu C. Fabrication of pea protein-tannic acid complexes: impact on formation, stability, and digestion of flaxseed oil emulsions. Food Chem. (2019) 310:125828.1–11. 10.1016/j.foodchem.2019.125828 31812319

[45] Chung C Sher A Rousset P McClements DJ. Impact of oil droplet concentration on the optical, rheological, and stability characteristics of O/W emulsions stabilized with plant-based surfactant: potential application as non-dairy creamers. Food Res Int. (2018) 105:913–9. 10.1016/j.foodres.2017.12.019 29433288

[46] Paglarini CS Figueiredo Furtado G de Biachi JP Vidal VAS Martini S Forte MBS. Functional emulsion gels with potential application in meat products. J Food Eng. (2018) 222:29–37. 10.1016/j.jfoodeng.2017.10.026

[47] Marto J Gouveia L Jorge IM Duarte A Gonçalves LM Silva SMC. Starch-based Pickering emulsions for topical drug delivery: a QbD approach. Colloids Surf B Biointerfaces. (2015) 135:183–92. 10.1016/j.colsurfb.2015.07.024 26263210

[48] Pang B Zhang H Schilling M Liu H Wang X Rehfeldt F. High-internal-phase pickering emulsions stabilized by polymeric dialdehyde cellulose-based nanoparticles. ACS Sustain Chem Eng. (2020) 8:7371–9. 10.1021/acssuschemeng.0c01116

[49] Yi J Gao L Zhong G Fan Y. Fabrication of high internal phase Pickering emulsions with calcium-crosslinked whey protein nanoparticles for β-carotene stabilization and delivery. Food Funct. (2020) 11: 768–78. 10.1039/C9FO02434D 31917381

[50] Liu X Guo J Wan ZL Liu Y Ruan QJ Yang XQ. Wheat gluten-stabilized high internal phase emulsions as mayonnaise replacers. Food Hydrocoll. (2018) 77:168–75. 10.1016/j.foodhyd.2017.09.032

[51] Campanella OH Dorward NM Singh H. A study of the rheological properties of concentrated food emulsions. J Food Eng. (1995) 25:427–40. 10.1016/0260-8774(94)00000-Y

[52] Liu F Tang CH. Soy glycinin as food-grade Pickering stabilizers: part. I. Structural characteristics, emulsifying properties and adsorption/arrangement at interface. Food Hydrocoll. (2016) 60:606–19. 10.1016/j.foodhyd.2015.04.025

[53] Caro AL Niño MRR Patino JMR. The effect of pH on structural, topographical, and rheological characteristics of β-casein–DPPC mixed monolayers spread at the air–water interface. Colloids Surf . (2009) 332:180–91. 10.1016/j.colsurfa.2008.09.020

[54] Lucero A Rodriguez Nino MR Gunning AP Morris VJ Wilde PJ Rodríguez Patino JM. Effect of hydrocarbon chain and pH on structural and topographical characteristics of phospholipid monolayers. J Phys Chem B. (2008) 112:7651–61. 10.1021/jp8013157 18517243

[55] Dickinson E. Milk protein interfacial layers and the relationship to emulsion stability and rheology. Colloids Surf B Biointerfaces. (2001) 20:197–210. 10.1016/S0927-7765(00)00204-6 11172975

[56] Muarry BS Dickibson E. Interfacial rheology and the dynamic properties of adsorbed films of food proteins and surfactants. Food Sci Technol Int. (1996) 2:131–45. 10.3136/fsti9596t9798.2.13130469122

[57] Li R Peng S Zhang R Dai T Fu G Wan Y. Formation and characterization of oil-in-water emulsions stabilized by polyphenol-polysaccharide complexes: tannic acid and β-glucan. Food Res Int. (2019) 123:266–75. 10.1016/j.foodres.2019.05.005 31284976

[58] Liu YK Yan CH Chen J Wang Y Liang R Zou L. Enhancement of beta-carotene stability by encapsulation in high internal phase emulsions stabilized by modified starch and tannic acid. Food Hydrocoll. (2020) 109:106083. 10.1016/j.foodhyd.2020.106083

[59] Chen S Zhang L. Casein nanogels as effective stabilizers for pickering high internal phase emulsions. Colloids Suf . (2019) 579 123662. 10.1016/j.colsurfa.2019.123662

[60] Li M Zou L McClements DJ Liu W. One-step preparation of high internal phase emulsions using natural edible Pickering stabilizers: Gliadin nanoparticles/gum Arabic. Food Hydrocoll. (2020) 100:105381. 10.1016/j.foodhyd.2019.105381

[61] Kim H Decker E Julianmcclements D. Preparation of multiple emulsions based on thermodynamic incompatibility of heat-denatured whey protein and pectin solutions. Food Hydrocoll. (2006) 20:586–95. 10.1016/j.foodhyd.2005.06.007

